# Supplementary figures and images for: Conditional U1 Gene Silencing in Toxoplasma gondii
Source: PLoS One. 2015 Jun 19;10(6):e0130356. doi: 10.1371/journal.pone.0130356 (PMC4474610; doi:10.1371/journal.pone.0130356)

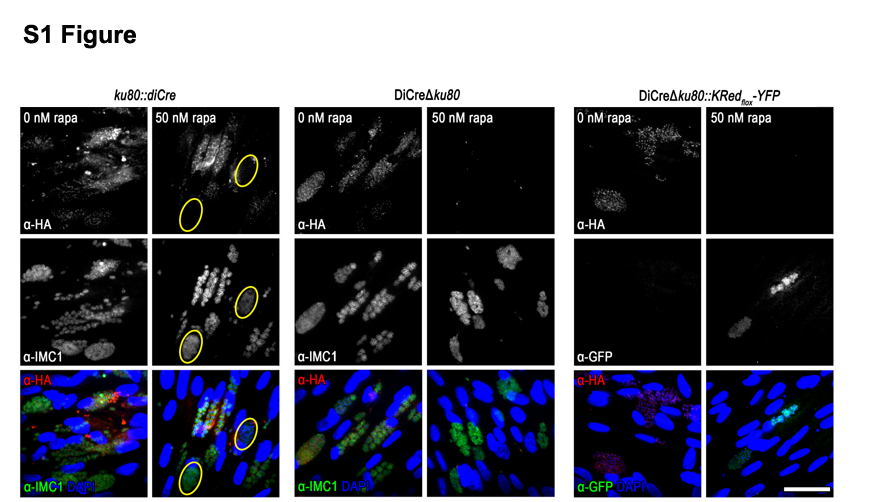

Supplement: S1 Fig — A dotted staining pattern for DrpC-HA was observed in all three recipient strains. DiCreΔku80 and DiCre ku80::KRed flox -YFP parasites show efficient rapamycin dependent drpC silencing. In contrast, in ku80::diCre parasites only in a few drpC silenced vacuoles (encirceled in yellow) were observed. (TIF) [file pone.0130356.s001.tif]
